# Effects of Cu/SnAgCu Powder Fraction and Sintering Time on Microstructure and Mechanical Properties of Transient Liquid Phase Sintered Joints

**DOI:** 10.3390/ma17092004

**Published:** 2024-04-25

**Authors:** Dinh-Phuc Tran, Yu-Ting Liu, Chih Chen

**Affiliations:** Department of Materials Science and Engineering, National Yang Ming Chiao Tung University, Hsinchu 300093, Taiwan; trandinhphuc1508@gmail.com (D.-P.T.); sophia840117@gmail.com (Y.-T.L.)

**Keywords:** thermal interface materials, intermetallic compounds, thermal cycling test, high-temperature storage, shear strength

## Abstract

The effects of the sintering duration and powder fraction (Ag-coated Cu/SnAgCu) on the microstructure and reliability of transient liquid phase sintered (TLPS) joints are investigated. The results show that two main intermetallic compounds (IMCs, Cu_6_Sn_5_ and Cu_3_Sn) formed in the joints. The Cu_6_Sn_5_ ratio generally decreased with increasing sintering time, Cu powder fraction, and thermal treatment. The void ratio of the high-Cu-fraction joints decreased and increased with increasing sintering and thermal stressing durations, respectively, whereas the low-Cu-fraction counterparts were stable. We also found that the shear strength increased with increasing thermal treatment time, which resulted from the transformation of Cu_6_Sn_5_ and Cu_3_Sn. Such findings could provide valuable information for optimizing the TLPS process and assuring the high reliability of electronic devices.

## 1. Introduction

In the electronics industry, transient liquid phase sintering (TLPS) is a common method for bonding electronic components at a low processing temperature and pressure. During sintering processes, the metal with a low melting point covers its high-temperature counterparts [1,2,3,4,5]. Liquid–solid diffusion then occurs, leading to the formation of intermetallic compounds (IMCs). TLPS of a Ag and Sn powder mixture was conducted to bond Cu substrates and metalized Si chips [2]. Cu–Sn and Ag_3_Sn IMCs were found in the sintered joint. The microstructure evolution and mechanical strength of the Ag/Sn/Cu TLPS joints during aging were investigated [3]. It was found that almost no deterioration of the joints occurred during the aging tests. Cu–Sn and Ag_-_Sn IMCs were also observed [3]. Recently, various TLPS metallic couples have been proposed [6,7,8,9,10,11], but among those systems, Cu-Sn-Ag [12,13] and Cu-Sn [14,15,16,17] are the most popular solders. These solder structures can ensure good thermal conductivity and high mechanical strength [12,13,14,15,16,17]. However, the reliability issues related to the void formation and brittle nature of such solders are still in need of consideration.

Additionally, the establishment of atomic-level bonds during liquid–solid diffusion is a slow process compared to the rapid melting and solidification via traditional soldering. TLPS thus is accompanied with a long bonding time [18,19,20]. Many approaches have been employed to accelerate the sintering time such as high-temperature/pressure bonding [19,21,22], incorporation of reactive additives [23,24,25,26], surface modification [27,28], and electric/laser-assisted bonding [29,30,31,32,33,34]. For instance, Ramli et al. employed TiO_2_ particles to reduce the thickness of the interfacial IMCs for the enhancement of the shear strength and hardness of the solder joints [24]. Bhogaraju et al. used a novel approach of surface modifications to enhance the sinterability of the Cu flakes, which facilitated the formation of a dense and close-packed structure [26]. An electroless nickel, palladium, and immersion gold multilayer was also developed to enhance the bonding strength of BiTe-based modules [28]. A laser-assisted bonding technique was applied to minimize the thermal damage to the electronic components [31]. A thinner IMC layer formed, and the void volume was lower compared to that of the solders produced by a traditional reflow process. However, this can increase the fabrication expenditure and generate challenges in achieving a homogeneous mixture of the sintered pastes, limiting their commercialization.

In the semiconductor industry, Cu and Sn are widely considered as low-cost metals in electronics [35,36]. The diffusion reactions forming IMCs can occur at a relatively low temperature [37,38,39,40,41]. Such characteristics are beneficial for electronic packaging technology, suppressing the risk of thermal damage to sensitive electronic devices. During Cu-Sn sinter bonding, Cu_6_Sn_5_ and Cu_3_Sn IMCs commonly form in the sintered joints [42,43,44]. Such a process leads to the complete formation of a liquid phase of the low-melting-temperature metal and IMC transformation. However, the sintered metals may remain, which can degrade the bonding strength of the solder joints. To obtain the desired bonding strength and fully sintered features, it is crucial to explore the sintering parameters and material compositions.

The Cu-SnAgCu sintered joints can be adopted as the die attach layer for high-power device packaging. Currently, pastes of Ag particles are commonly used for this layer [45,46,47,48]. Sputtered (111)-oriented Ag films are also under investigation [49,50,51,52]. Cu-Sn powders/pastes may also serve as die attach materials because the Cu-Sn reaction rate is very fast and its thermal conductivity is good. However, studies on the application of sintered Cu-SnAgCu as a die attach layer and its bonding reliability are limited. In this study, fully sintered joints were fabricated, and the effects of the Cu/Sn powder fraction and sintering time on the microstructures and bonding strength of the sintered joints were then investigated. The reliability was also correlated with the formation of voids and IMCs using high-temperature storage (HTS) and thermal cycling tests (TCTs). This study offers a full understanding of the bonding behaviors of different Cu powder fractions and sintering durations, which is essential for the optimization of the fabrication process and assuring the good reliability of electronic interconnects for packaging applications.

## 2. Materials and Methods

**Figure 1:**
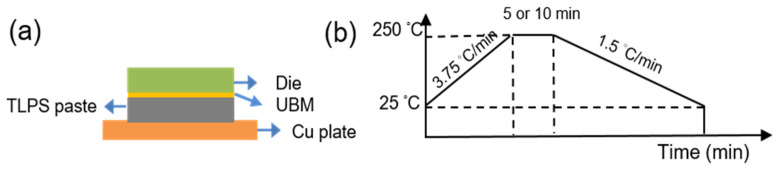
Schematic of the (**a**) TLPS joints and (**b**) sintering profile. The sintering temperature (250 °C) was held for 5 or 10 min.

Ag-coated Cu (Cu_94.0_Ag_6.0_) and Sn-Ag-Cu solder (Sn_96.5_Ag_3.0_Cu_0.5_) powders with diameters of 0.8~3.5 μm and 2~11 μm, respectively, were mixed with specified weight fractions (50/50 and 40/60). During sinter bonding, the complete formation of the liquid phase of the low-melting-temperature metal and IMC transformation are achieved. With a high ratio of Cu powders, Cu powders may abundantly remain, which significantly degrades the bonding strength of the solder joints. Therefore, the weight fractions of Cu powders (50/50 and 40/60) were selected to explore the effects of sintering duration and powder fraction on the microstructure and reliability of the sintered joints. In electronic packaging, the sintering time is typically a few minutes at temperatures ranging from 200 °C to 250 °C. Such a sintering duration can establish atomic-level bonds during liquid–solid diffusion processes. Thus, in this study, the sintering temperature of 250 °C was maintained for 5 or 10 min. They were then blended at a weight fraction of 85/15 to form a solder paste for wetting. The TLPS pastes were then spread on various dies using a stainless steel sheet and heated in an oven for 30 min. This pre-baking step served to volatilize and remove the flux from the pastes. The schematics of the TLPS joints and sintering profile are shown in Figure 1. The top dies consisted of a Cu under bump metallization (UBM), which was sputtered on the Si substrates for connecting the dies with the solder, as shown in Figure 1a. The samples were then hot-pressed to bond with Cu plates with a pressure of 12.68 MPa. The sintering temperature was increased to 250 °C with a heat rate of 3.75 °C/min and maintained at that temperature for a certain time (5 min or 10 min). After the sintering, the samples were then cooled down to room temperature with a cooling rate of 1.5 °C/min.

The reliability of the sintered joints was assessed by HTS and TCTs. The HTS tests were conducted at 150 °C for 1000 h, whereas the TCTs were performed at a temperature range of −40 to 125 °C for 1000 cycles. The heating and cooling rates were 15 °C/min. In addition, the bonding strength of the samples before and after the HTS and TCTs was characterized by shear tests. These tests aimed to provide insights into the reliability performance and potential microstructural changes in the sintered joints under various environmental factors that exist in electronic devices. The joints were then cross-sectioned and analyzed by an optical microscope (OM). Scanning electron microscopy (SEM, JSM-7800F, JEOL Ltd., Tokyo, Japan) and energy-dispersive X-ray (EDX, JSM-7800F, JEOL Ltd., Tokyo, Japan) analyses were also performed to characterize the microstructures of the initial powders and IMC compositions of the sintered joints.

## 3. Results and Discussion

**Figure 2:**
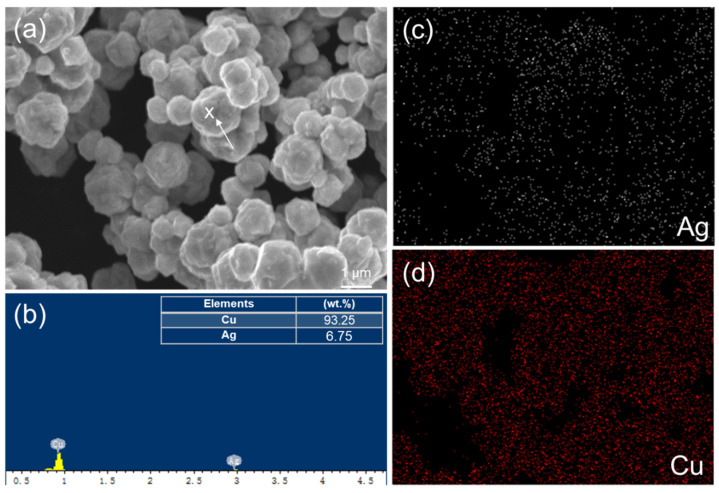
(**a**) SEM image and EDX analysis of the Ag-coated Cu (Cu_94.0_Ag_6.0_) powders. (**b**) Typical EDX spectrum taken from the white spot labeled as “X” in (**a**). Elemental mappings of (**c**) Ag, (**d**) Cu.

**Figure 3:**
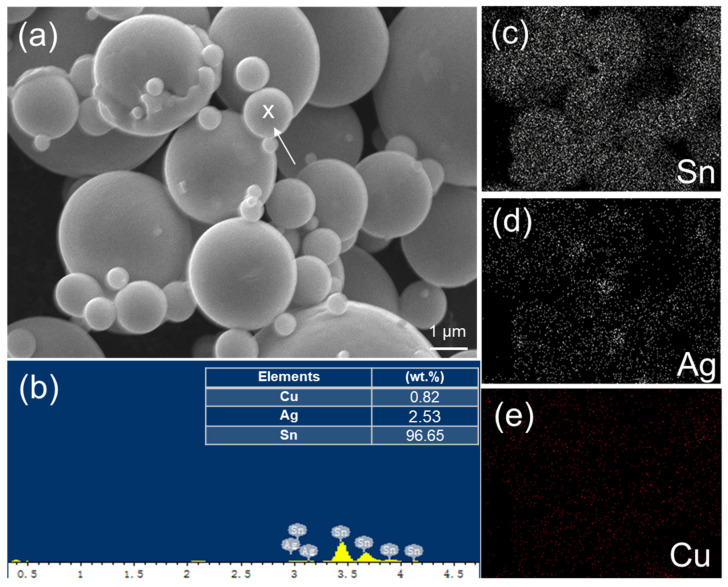
(**a**) SEM image and EDX analysis of the Sn_96.5_Ag_3.0_Cu_0.5_ powders. (**b**) Typical EDX spectrum taken from the white spot labeled as “X” in (**a**). Elemental mappings of (**c**) Sn, (**d**) Ag, and (**e**) Cu.

The microstructures of two initial powders were analyzed using SEM and EDX. The SEM images and EDX spectra of the Ag-coated Cu (Cu_94.0_Ag_6.0_) and Sn96.5Ag3.0Cu0.5 powders are shown in Figure 2 and Figure 3, respectively. The size of the Ag-coated Cu powders ranged from 0.5 to 2.1 µm, whereas the Sn96.5Ag3.0Cu0.5 powders were spherical with a size range of ~0.2 to 5.2 µm. The former type of powder (Figure 2a) was more uniform than the latter (Figure 3a). Additionally, the EDX mappings show the uniform distributions of the elements in the powders and the high purity of the powders. As shown in Figure 2c,d, the Cu powders were dominant and uniformly embedded with Ag. The Sn_96.5_Ag_3.0_Cu_0.5 _powders mostly consisted of spherical Sn with some small amounts of Ag and Cu, as shown in Figure 3c–e. These powders were then mixed with different weight fractions and sintered to form TLPS joints.

**Figure 4:**
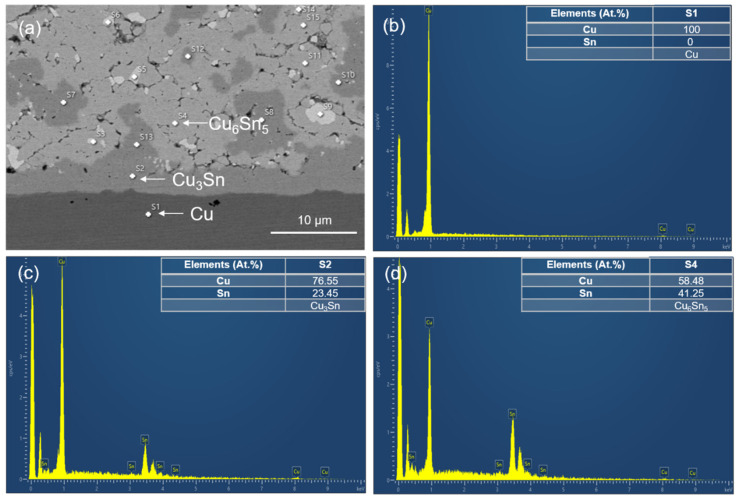
(**a**) SEM image and EDX analysis of the IMCs taken at the lower region of the joints sintered for 5 min from the 40%-Cu-fraction powders. (**b**–**d**) Typical EDX spectra showing the elemental composition in the white-dotted regions labeled as (s1, s2, and s4).

**Figure 5:**
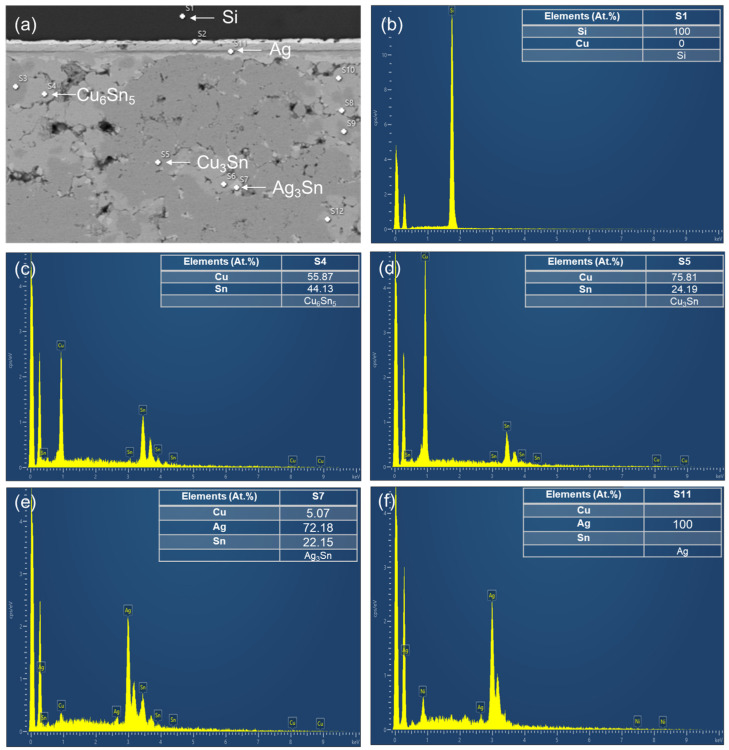
(**a**) SEM image and EDX analysis of the IMCs taken at the lower region of the joints sintered for 5 min from the 50% Cu fraction powders. (**b**–**f**) Typical EDX spectra showing the elemental composition in the white-dotted regions labeled as (s1, s4, s5, s7, and s11).

**Figure 6:**
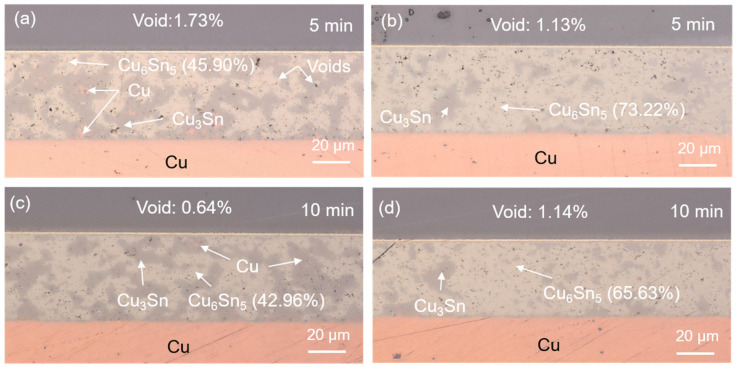
Cross-sectional OM images of the solder joints sintered with (**a**,**c**) 50 wt% and (**b**,**d**) 40 wt% Cu powders for 5 and 10 min. The remaining Cu powders and Cu_6_Sn_5_ and Cu_3_Sn IMCs are represented by the orange and light and dark grays, respectively.

**Figure 7:**
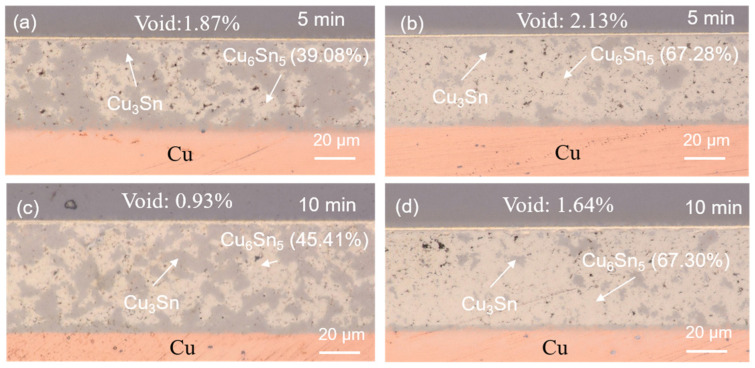
Cross-sectional OM images of the sintered joints after 500 h of HTS tests. The joints were sintered with (**a**,**c**) 50 wt% and (**b**,**d**) 40 wt% Cu powders for 5 and 10 min.

The SEM images of the upper and lower locations of the sintered joints with their elemental compositions are shown in Figure 4 and Figure 5. Most of the Cu powders reacted with the SnAgCu solders. Two main IMCs (Cu_6_Sn_5_ and Cu_3_Sn) and voids were detected in the sintered joints. We also found the significant IMC transformation of Cu_6_Sn_5_ to Cu_3_Sn as the sintering time was extended. The cross-sectional OM images of the sintered joints are shown in Figure 6. Voids and IMCs can be clearly observed. The ratios of the voids and IMCs observed in the cross-sections were acquired using image processing software that the OM was equipped with. It can be seen that the voids are randomly distributed in the sintered joints. The voids formed in the joints (Figure 6a,b) sintered for 5 min with Cu powder fractions of 50% and 40% accounted for 1.72% and 1.13%, respectively. As the sintering time was prolonged to 10 min, the ratio of the voids in the 50%-Cu-fraction joints significantly decreased to 0.64% (Figure 6c). However, no obvious difference in the void ratio was found in the joints sintered with 40% Cu powders (Figure 6d). Additionally, the Cu_6_Sn_5_ and Cu_3_Sn IMCs could be clearly observed in the OM images (Figure 7). The Cu_6_Sn_5_ and Cu_3_Sn IMCs are denoted by the light and dark grays, respectively. We also detected some remaining Cu powder (orange) in the joints after the sintering (Figure 7a,c). The remaining Cu powders in the joints indicated the incomplete sintering of the powders with a 50% Cu fraction. However, no Cu powders were found in the 40%-Cu-fraction joints.

The Cu_6_Sn_5_ IMC accounted for 45.90% of the 50%-Cu-fraction joints sintered for 5 min (Figure 6a). This proportion markedly increased to 73.22% as the weight fraction of the Cu powders was reduced to 40% (Figure 6b). The ratio of the Cu_6_Sn_5_ IMC slightly decreased as the sintering time was prolonged to 10 min (Figure 6c,d). Generally, the interaction between Cu and Sn, forming the IMCs, increases with the increase in the reaction time [53,54]; however, the opposite trend was found here. This could be attributed to the effect of the Cu/Sn fraction during sintering. The variations in the initial Cu/Sn amounts could result in different formation mechanisms of the IMCs, hindering the increase in Cu_6_Sn_5_, but, rather, benefitting the increase in Cu_3_Sn [55,56,57]. After the sintering reaction, almost all of the Sn atoms reacted with Cu to form Cu_6_Sn_5_ and Cu_3_Sn. Yet, the supply of Cu could have been from the unreacted Cu powders or from the Cu substrate, according to the reaction below:

Cu_6_Sn_5_ + 9Cu → 5Cu_3_Sn (1)

The Cu_6_Sn_5_ IMCs prefer to transform into Cu_3_Sn IMCs after the SnAgCu solders are consumed completely and the supply of Cu is still abundant.

**Figure 8:**
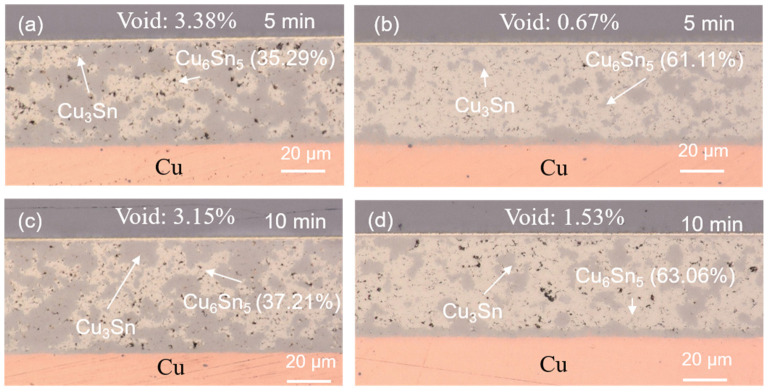
Cross-sectional OM images of the sintered joints after 1000 h of HTS tests. The joints were sintered with (**a**,**c**) 50 wt% and (**b**,**d**) 40 wt% Cu powders for 5 and 10 min.

**Figure 9:**
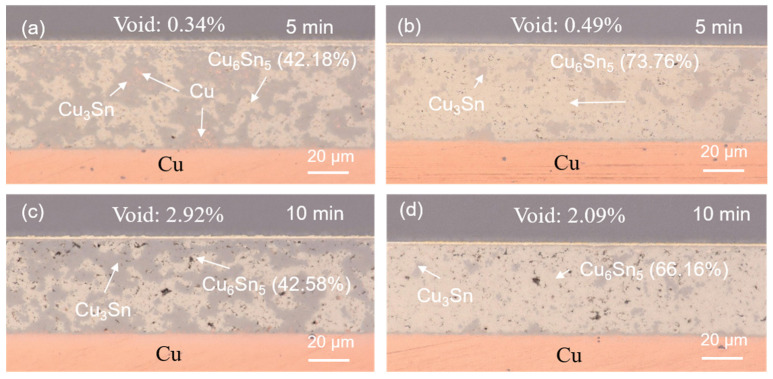
Cross-sectional OM images of the sintered joints after 500 thermal cycles. The joints were sintered with (**a**,**c**) 50 wt% and (**b**,**d**) 40 wt% Cu powders for 5 and 10 min.

**Figure 10:**
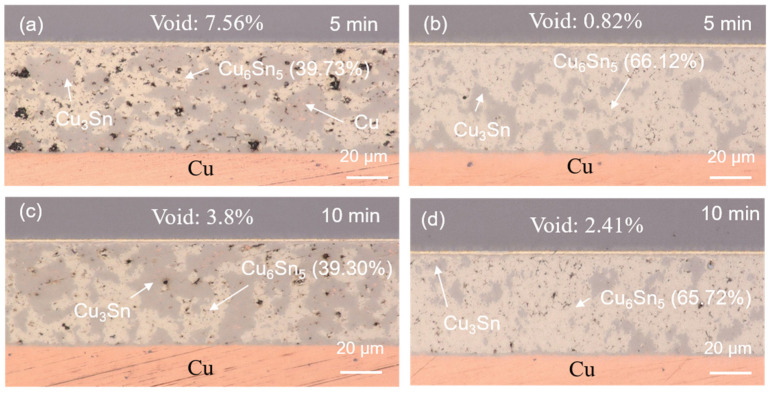
Cross-sectional OM images of the sintered joints after 1000 thermal cycles. The joints were sintered with (**a**,**c**) 50 wt% and (**b**,**d**) 40 wt% Cu powders for 5 and 10 min.

The reliability evaluations on the sintered joints were performed using HTS and TCT tests. The cross-sectional OM images of the sintered joints after the reliability tests are shown in Figure 7, Figure 8, Figure 9 and Figure 10. No Cu powders were found in the joints after 500 h or 1000 h of HTS tests (Figure 7 and Figure 8). This indicated that the remaining Cu powders completely interacted with the solders and fully transformed to IMCs after the HTS tests. The thermal energy of such a high temperature could facilitate Cu/Sn diffusion and fulfill the sintering process, resulting in the complete transformation of the IMC. However, some Cu powders still remained in the 50%-Cu-fraction joints when subjected to thermal cycling, as shown in Figure 9a and Figure 10a. The energy of the thermal cycling was insufficient to fully transform the remaining Cu powders in the 50%-Cu-fraction joints.

**Figure 11:**
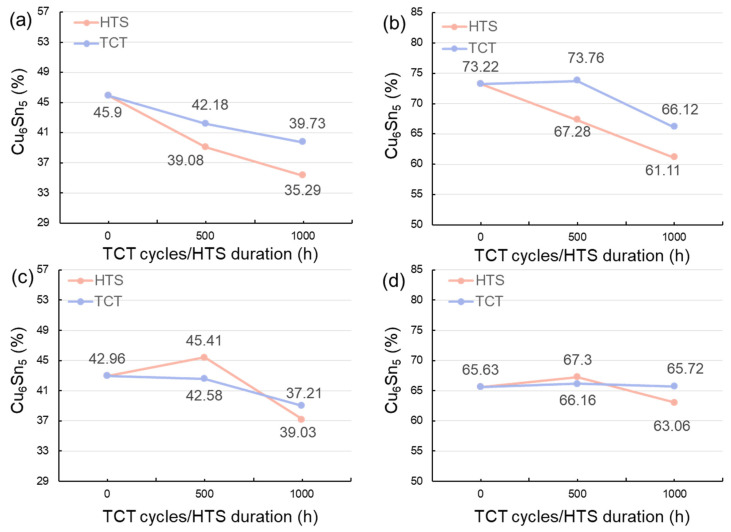
Ratios of Cu_6_Sn_5_ IMC formed in the sintered joints before and after HTS and TCTs. The joints were sintered with (**a**,**c**) 50 wt% and (**b**,**d**) 40 wt% Cu powders for 5 and 10 min.

Additionally, it can be seen that an IMC layer formed at the solder–Cu substrate interface during solid state aging (Figure 7, Figure 8, Figure 9 and Figure 10). The Cu_6_Sn_5_ IMCs near the interface preferred to transform into Cu_3_Sn IMCs, according to the reaction (1). The abundant supply of Cu could be from the Cu substrate. The systematic study of the IMC transformation at the solder–Cu substrate interface could be an interesting topic in the future. The ratios of the Cu_6_Sn_5_ IMC at some specific periods of the reliability tests are shown in Figure 11. It can be seen that the Cu_6_Sn_5_ ratio significantly decreased under HTS and TCTs, whereas the Cu_3_Sn ratio showed the opposite trend (Figure 11a,b). This indicated that the Cu_6_Sn_5_ IMC significantly transformed into the Cu_3_Sn IMC during the reliability tests. However, the IMC ratios in the joints sintered for a longer time (10 min) remained almost unchanged during the HTS and TCTs (Figure 11c,d). The Cu_6_Sn_5_ IMC almost reached its equilibrium state. The results suggest that the longer sintering time stabilized the sintered joints, making them less susceptible to microstructural and mechanical changes under thermal stress.

**Figure 12:**
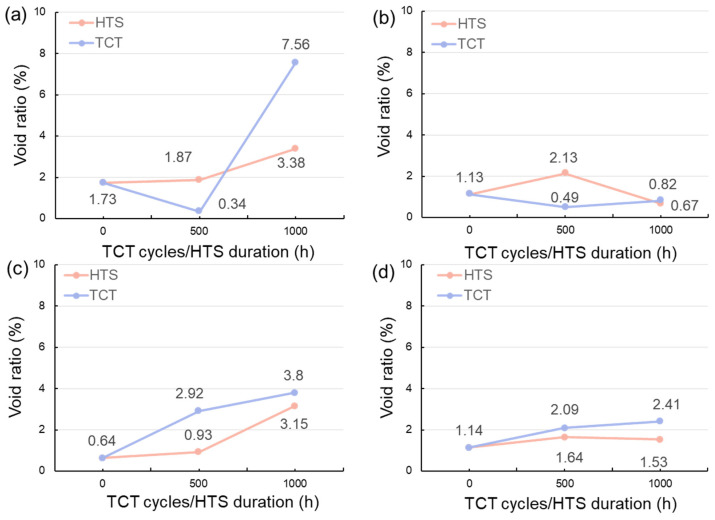
Void ratios of the sintered joints before and after HTS and TCTs. The joints were sintered with (**a**,**c**) 50 wt% and (**b**,**d**) 40 wt% Cu powders for 5 and 10 min.

The void ratios in the sintered joints after HTS and TCTs are shown in Figure 12. It can be seen that the void ratio in the 50%-Cu-fraction joints significantly decreased (1.73 to 0.64%) as the sintering time was prolonged from 5 to 10 min (Figure 12a,c). However, the void ratio in the 40%-Cu-fraction joints did not obviously change with the sintering time (Figure 12b,d). Regarding the joints fabricated from the powders with a 50% fraction of Cu, the void ratio generally increased with increased HTS duration and thermal cycling cycles (Figure 12a,c). However, no obvious changes in the void ratios of the 40%-Cu-fraction joints were found. The results indicate that the formation of voids in the low-Cu-fraction joints was relatively stable and not affected by the thermal stressing. As aforementioned (Figure 6), some Cu powders remained in the 50%-Cu-fraction joints whereas no Cu powders were found in the 40%-Cu-fraction joints. The changes in the void ratios could be attributed to the presence of the remaining Cu powders subjected to thermal stressing.

It is noted that the molar volumes of the reactant elements (Cu and Sn) and the formed IMCs (Cu_3_Sn and Cu_6_Sn_5_) are 7.1, 16.1, 34.8, and 117.7 cm^3^/mol, respectively. The specific volume shrinkage after the Cu-Sn soldering reactions (6Cu + 5Sn → Cu_6_Sn_5_; 3Cu + Sn → Cu_3_Sn) could be estimated as 4.4% and 7.1%, respectively. This indicates that the shrinkage of the joints with a higher amount of the resultant Cu_3_Sn IMC was larger than that with the Cu_6_Sn_5_. Additionally, according to Equation (1), one mole of Cu_6_Sn_5_ (117.7 cm^3^) reacts with nine moles of Cu (63.9 cm^3^) to form five moles of Cu_3_Sn (174.0 cm^3^) when the Cu powders are abundant. This leads to volume shrinkage (4.1%). During the HTS and TCTs, the IMC transformation from Cu_6_Sn_5_ to Cu_3_Sn could result in the formation of a porous-type Cu_3_Sn IMC [57] and thus lead to the obvious increase in the void ratios of the 50%-Cu-fraction joints, as shown in Figure 11a,c and Figure 12a,c.

**Figure 13:**
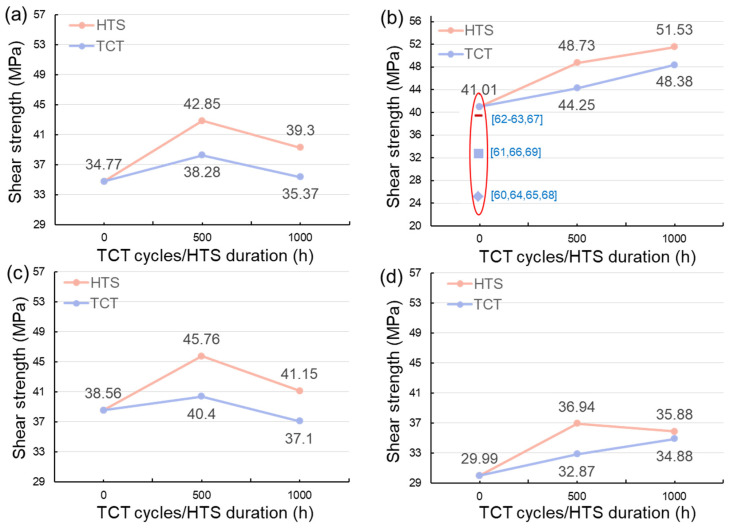
Shearing strength of the sintered joints before and after HTS and TCTs. The joints were sintered with (**a**,**c**) 50 wt% and (**b**,**d**) 40 wt% Cu powders for 5 and 10 min.

**Figure 14:**
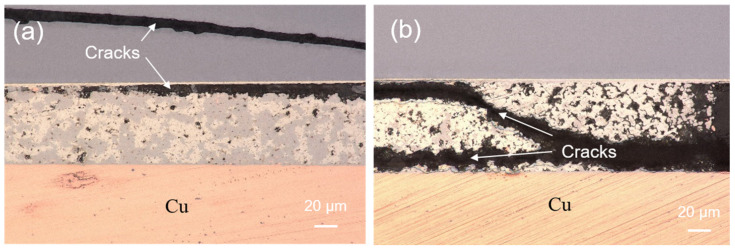
Cross-sectional OM images showing fractures at the edges of the joints sintered with (**a**) 50 wt% and (**b**) 40 wt% Cu powders for 5 and 10 min, respectively, after subjecting to 1000 thermal cycles.

Shear tests were also performed to evaluate the bonding strength of the sintered joints before and after the reliability tests. The shearing strength of the sintered joints before and after the HTS and TCTs are shown in Figure 13. We found that the shear strength of the 50%-Cu-fraction joints increased with increasing sintering time (Figure 13a,c), whereas an opposite trend were observed in the 40%-Cu-fraction joints (Figure 13b,d). This could be attributed to the differences in the IMC and void ratios of the sintered joints. The void ratios in the 50%-Cu-fraction joints significantly decreased (Figure 6a,c) as the sintering time was extended, favoring the bonding strength of the joints. Note that the IMCs formed in the sintered joints are naturally brittle. Thus, excessive IMC formation could negatively weaken the joint strength over prolonged sintering (Figure 6b,d). Additionally, we found that the shear strength of the joints generally increased with increasing thermal stress (Figure 13). As shown in Figure 11, the ratio of the Cu_6_Sn_5_ IMC decreased during the reliability tests due to the transformation to the Cu_3_Sn IMC. It has been reported that the mechanical strength of the Cu_3_Sn IMC is typically higher than that of its Cu_6_Sn_5_ counterpart [42,58,59]. Thus, a larger ratio of the Cu_3_Sn IMC could be attributed to the higher shear strength of the sintered joints. Additionally, we found some cracks at the edges of the joints that were sintered using 50 wt% (Figure 14a) and 40 wt% (Figure 14b) Cu powders for 5 and 10 min, respectively, after subjecting them to 1000 thermal cycles. No cracks were observed in the other joints. This indicated the weak strength of those joints under thermal cycling, as shown in Figure 6b,d. In addition, the shear strength of the joints was compared with that of the sintered joints reported in recent studies [60,61,62,63,64,65,66,67,68,69]. Clearly, the joints showed a high shear strength and good reliability, showing their potential for packaging applications.

## 4. Conclusions

In summary, the effects of the Cu powder fraction and sintering time on the void and IMC ratios of the sintered joints are analyzed. Some findings are drawn from the current study as follows:

During the sintering process, Sn reacted with Cu powders and transformed into two main (Cu_6_Sn_5_ and Cu_3_Sn) IMCs. The ratio of Cu_6_Sn_5_ formed decreased as the sintering time was extended. It significantly increased with decreasing Cu powder fraction. The changes in the initial Cu/Sn supply could cause the different formation mechanisms of the IMCs, suppressing the growth of the Cu_6_Sn_5_ IMC, but, rather, facilitating Cu_3_Sn growth.

Some remaining Cu powders were also found in the high-Cu-fraction joints, which indicated incomplete sintering. We found that the void ratio of the high-Cu-fraction joints markedly decreased with increasing sintering time. However, the void ratio in the low-Cu-fraction counterparts remained unchanged.

HTS and TCTs were also performed to evaluate the reliability of the joints. The void ratio of the high-Cu-fraction joints increased with increasing thermal stressing time, whereas the low-Cu-fraction ones were stable. The shear strength of all the sintered joints increased with increasing thermal treatment, which could be attributed to IMC transformation between Cu_6_Sn_5_ and Cu_3_Sn.

## Data Availability

Data are contained within the article.
